# Accretion of “young” Phanerozoic subcontinental lithospheric mantle triggered by back-arc extension—the case of the Ivrea-Verbano Zone

**DOI:** 10.1038/s41598-024-61763-3

**Published:** 2024-05-23

**Authors:** Abimbola C. Ogunyele, Alessio Sanfilippo, Vincent J. M. Salters, Mattia Bonazzi, Alberto Zanetti

**Affiliations:** 1 Department of Earth and Environmental Sciences, University of Pavia, Via Ferrata 1, 27100 Pavia, Italy; 2 CNR – Istituto Geoscienze e Georisorse, Via Ferrata 1, 27100 Pavia, Italy; 3 Department of Earth Sciences, Adekunle Ajasin University, PMB 001, Akungba-Akoko, Nigeria; 4 National High Magnetic Field Laboratory, Department of Earth, Ocean and Atmospheric Sciences, Florida State University, Tallahassee, FL 32310 USA

**Keywords:** Subcontinental Lithospheric Mantle, Paleozoic mantle accretion, Ivrea-Verbano Zone, Orogenic lherzolites, Phanerozoic regions, Back-arc extension, Geochemistry, Geodynamics, Petrology

## Abstract

The subcontinental lithospheric mantle (SCLM) beneath Phanerozoic regions is mostly constituted by fertile lherzolites, which sharply contrast with cratonic mantle made of highly-depleted peridotites. The question of whether this chemical difference results from lower degrees of melting associated with the formation of Phanerozoic SCLM or from the refertilization of ancient depleted SCLM remains a subject of debate. Additionally, the timing and geodynamic environment of accretion of the fertile SCLM in many Phanerozoic regions are poorly constrained. We here document new geochemical and Nd-Hf isotopic data for orogenic lherzolite massifs from the Ivrea-Verbano Zone (IVZ), Southern Alps. Even though a few Proterozoic Re depletion ages are locally preserved in these mantle bodies, our data reveal that the IVZ lherzolitic massifs were “recently” accreted to the SCLM in the Upper Devonian (ca. 370 Ma) during Pangea amalgamation, with a petrochemical evolution characterized by low-degree (~ 5–12%) depletion and nearly contemporaneous pervasive to focused melt migration. The lithospheric accretion putatively took place through asthenospheric upwelling triggered by Variscan intra-continental extension in a back-arc setting related to the subduction of the Rheic Ocean. We thus conclude that the fertile sections of Phanerozoic SCLM can be accreted during “recent” events of back-arc continental extension, even where Os isotopes preserve memories of melting events in much older times.

## Introduction

**Figure 1:**
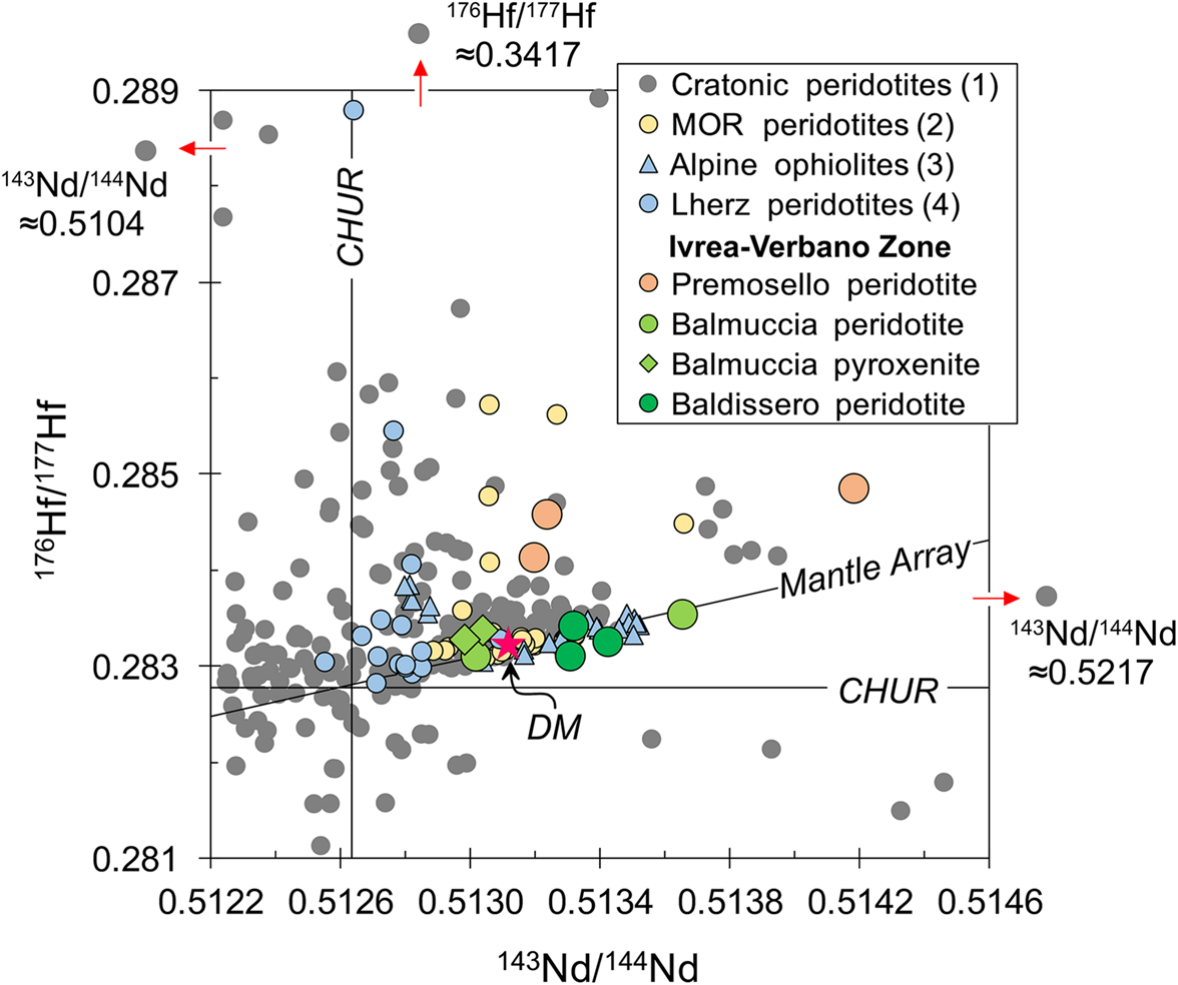
Present-day Nd-Hf diagram showing depletion signatures preserved in IVZ lherzolites and pyroxenites compared to (1) Cratonic peridotites; (2) Mid-Ocean Ridge (abyssal) peridotites; (3) Alpine ophiolites; and (4) Lherz orogenic peridotites. DM—Depleted Mantle^13^, CHUR—Chondritic Uniform Reservoir^14^. Plotted literature data are from the compilation of Ref ^5^.

The subcontinental lithospheric mantle (SCLM) beneath Phanerozoic (off-craton) terrains is made largely of peridotites having fertile compositions, contrary to cratonic areas where the lithosphere is dominantly formed by highly-depleted peridotites, more buoyant than the rest of the mantle and stable for billions of years^1^. This ancient history of depletion is revealed by the significantly higher than DM ^176^Hf/^177^Hf and ^143^Nd/^144^Nd compositions of mantle xenoliths from basalts and kimberlites in Precambrian terrains and Archean cratons^2^ (Fig. 1), which are often associated with unradiogenic ^187^Os/^188^Os preserving old Re depletion ages^3,4^. Such highly-depleted Nd-Hf isotope compositions suggest high time-integrated Sm/Nd and Lu/Hf ratios, evidence for ancient melting events. Still, extremely variable Nd-Hf isotopes characterize these cratonic peridotites, suggesting that the long-term preservation of depleted signatures can be overprinted by metasomatic episodes^5^. Highly-depleted Nd-Hf isotopic signatures, typical of cratonic areas, are nearly absent in subcontinental Phanerozoic lherzolite massifs which, consistently, are more fertile (i.e., lower Mg#, higher Al_2_O_3_, CaO, and Na_2_O) than the Archean peridotites^2^. Nonetheless, these lherzolitic massifs locally show Proterozoic to Archean Re depletion ages, suggesting that records of ancient melting episodes may be ubiquitously preserved in the upper mantle^4,6,7^. Hence, whether the fertile character of the Phanerozoic mantle reflects secular decreases in the average degree of melting associated with the formation of continental mantle^8,9^ or refertilization of ancient depleted SCLM portions^10–12^ is still strongly debated^5–7^. Moreover, the timing and environment of accretion of the SCLM in many Phanerozoic regions are open questions.

We here show that coupled Nd-Hf isotopes on residual to melt-reacted peridotites and associated pyroxenites from the Ivrea-Verbano Zone (IVZ) lherzolitic mantle massifs place fundamental constraints on the depletion signatures, timing, mechanism and geodynamic environment of accretion of Phanerozoic SCLM. Despite a few Re depletion ages extending up to 1.6 Ga^7^, we show that the IVZ fertile mantle lithosphere was accreted in the Paleozoic at ca. 370 Ma, during a process of intra-continental extension in a back-arc setting where low-degree (~ 5–12%) melting, pervasive metasomatism and pyroxenites segregation occurred almost synchronously. Rather than being a piece of cratonic mantle reworked during more recent tectonic cycles, we here document that the fertile SCLM beneath many Phanerozoic regions can be produced in “recent” times.

### Subcontinental Lithospheric Mantle in the Ivrea-Verbano Zone

The Ivrea-Verbano Zone (IVZ) is the westernmost sector of the Southern Alps and represents a continuously exposed section of lower to intermediate crust^15^. The IVZ consists of (i) a Variscan poly-metamorphic amphibolite- to granulite-facies volcano-sedimentary sequence representing the crystalline basement of the Adriatic Plate^16^; (ii) the Mafic Complex, an igneous complex formed by mantle-derived melts of upper carboniferous–lower permian age (314–282 Ma)^17,18^; and (iii) several lens-like peridotite bodies, which mostly have a mantle origin and differ in composition, degree of depletion and metasomatic overprint^19–22^.

Samples were selected from the largest spinel-facies lherzolite bodies, i.e., the Balmuccia, Baldissero and Premosello massifs, located in the central and southern sectors of IVZ (see Supplementary Fig. S1 in Supplementary Information). The Balmuccia and Baldissero massifs comprise mainly fresh spinel lherzolites (with ~ 10–15 vol. % clinopyroxene) and subordinate harzburgites and replacive dunites which, in Balmuccia, are cut by several generations of websterites, Cr-diopside and Al-augite pyroxenites^20,23–26^ (Supplementary Figs. S2–S3). The poorly-known Premosello massif consists of clinopyroxene-poor (~ 5 vol. % cpx) spinel lherzolite and minor replacive dunites also cut by Cr-diopside and Al-diopside pyroxenites^27^ (Supplementary Fig. S4). Previous geochemical data indicate that most IVZ lherzolites underwent variable extent of melt extraction and melt migration^7,20^. Available Re depletion ages (T_RD_) are predominantly Paleozoic with peak ages around 350–500 Ma, although a few Proterozoic depletion ages (up to 1.6 Ga) are locally preserved^7,25^. Consistently, Sm–Nd pseudo-isochron obtained from clinopyroxene separates of Baldissero peridotite furnished 378 ± 48 Ma^28^. Considering samples from both Baldissero and Balmuccia, Sm–Nd isotopes on clinopyroxene separates yield a pseudo-isochron at 390 Ma^29^.

The timing and mechanisms of emplacement of the IVZ mantle peridotites at lower crustal levels is still a matter of debate. Field and structural relationships suggest that some of the mantle peridotite bodies (e.g., the Balmuccia peridotite) were emplaced at crustal levels at the end of the Variscan orogeny^25^. Alternative hypotheses involve emplacement at crustal levels at the onset of the Mesozoic extensional regime, or tectonic addition to accretionary wedges of Paleozoic subduction zones^20^. Independent of the timing of crustal exhumation, recent gravimetric and seismic data converge to indicate that high-density rocks occur very close to the surface near the Insubric Line^30,31^, thus supporting the possibility that some of the mantle peridotite bodies (e.g., the Balmuccia peridotite) may be a direct expression of the underlying SCLM.

### New sample collection and analytical results

In this contribution, fresh and representative samples of spinel lherzolites were collected from the Balmuccia, Baldissero and Premosello massifs for mineral chemistry and Nd-Hf isotope systematics. Samples of the first generation of Cr-diopside pyroxenites cross-cutting the Balmuccia peridotite were also collected and investigated. Where possible, the lherzolite samples were collected far away (at least 50 cm) from other typologies of pyroxenites and gabbroic intrusives, although the pyroxenite trails along the tectonitic fabric in Premosello peridotite hampered the collection of samples far from Cr-diopside veins. Balmuccia and Baldissero peridotites show protogranular to weakly foliated textures (Supplementary Figs. S2–S3), whereas Premosello peridotite has a porphyroclastic texture defined by large, kinked crystals of olivine, orthopyroxene and clinopyroxene embedded within relatively finer grained crystals of similar minerals and spinel (Supplementary Fig. S4). Accessory amounts of Ti-rich amphibole and sulphides occur in all the three peridotite bodies.

**Figure 2:**
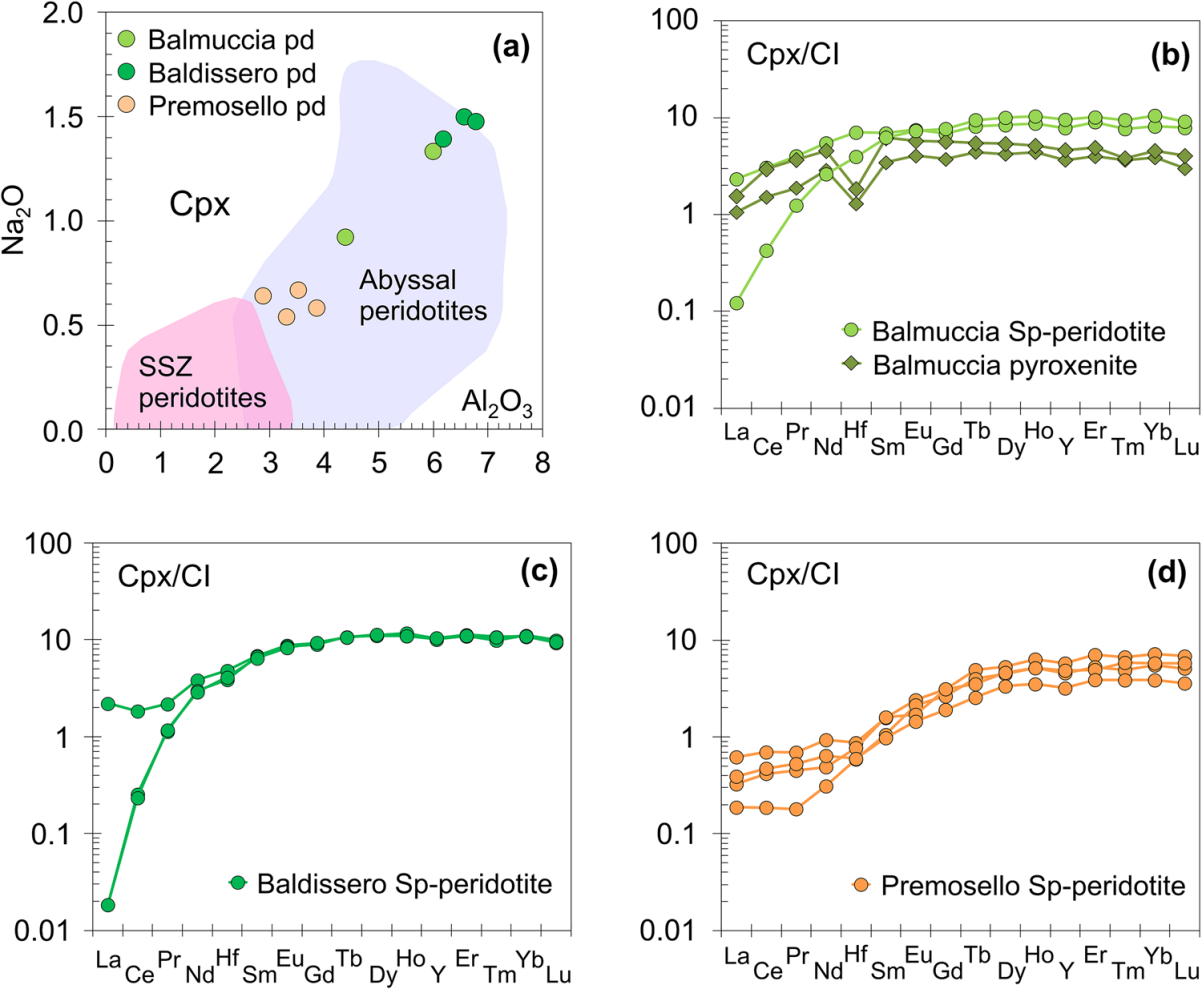
Plots of (**a**) Na_2_O (wt.%) versus Al_2_O_3_ (wt.%) in clinopyroxenes, and (**b**–**d**) CI-chondrite normalized REE–Hf patterns of clinopyroxenes from the studied IVZ peridotite and pyroxenite samples. pd—peridotite, SSZ—suprasubduction zone. CI-chondrite composition from Ref ^32^.

Mineral compositions distinguish the three peridotite bodies, with olivine, clinopyroxene (cpx) and orthopyroxene (opx) from the Premosello peridotite having the most refractory characters and highest Mg# (see Supplementary Table S1). Low Al_2_O_3_ and Na_2_O in cpx and high Cr# in spinel also characterize the Premosello peridotite compared to those from Balmuccia and Baldissero (Fig. 2a). In term of trace elements (Supplementary Table S2), cpx from Premosello peridotite show the lowest M-HREE and Hf contents, with REE patterns characterized by gradual decrease in chondrite-normalized concentrations from Lu to Sm, and nearly flat patterns from Nd to La [(Ce/Yb)_N_ = 0.03–0.12]. On the other hand, Balmuccia and Baldissero peridotites cpx REE patterns are more variable, ranging from samples having gradual decrease from Lu to La and marked LREE depletion [(Ce/Yb)_N_ = 0.02] to samples only slightly LREE-depleted [(Ce/Yb)_N_ = 0.4]. Cpx from the Balmuccia pyroxenites show REE patterns similar to cpx of the least depleted host peridotite, having slight depletions in LREE [(Ce/Yb)_N_ = 0.4–0.7] and distinct negative Hf anomaly. Notably, the pyroxenite cpx has enrichments in MREE with respect to HREE [(Sm/Yb)_N_ = 0.9–1.4], a feature not seen in the peridotites (Fig. 2b–d).

The present-day Nd-Hf isotopic compositions of cpx further confirm the difference between the three peridotite bodies (Supplementary Table S3). Balmuccia and Baldissero peridotites cpx display large variations in ^143^Nd/^144^Nd and ^176^Hf/^177^Hf and plot along the mantle Nd-Hf isotope array (Fig. 1). On close inspection, the strongly LREE-depleted Balmuccia and Baldissero peridotites cpx show radiogenic ^143^Nd/^144^Nd compositions, whereas the Balmuccia sample having high LREE has ^143^Nd/^144^Nd lower than Depleted Mantle (DM) composition^13^. Premosello peridotite cpx, instead, exhibits wider range in Nd-Hf isotope ratios, with Nd ranging from DM-like values towards highly radiogenic ^143^Nd/^144^Nd (up to 0.514181), and coupled with highly radiogenic Hf isotope compositions (^176^Hf/^177^Hf from 0.284139 to 0.284859) (Fig. 1). The three Premosello peridotite samples plot well above the mantle Nd-Hf isotope array, having Hf isotopes more radiogenic than most of the orogenic peridotites, and approaching the compositions of some abyssal peridotites^33,34^. Clinopyroxenes from the Balmuccia pyroxenites show Nd-Hf isotope ratios of 0.512982–0.513038 and 0.283284–0.283381, similar to the Balmuccia peridotite with high LREE contents and isotopically less depleted compared to the other peridotites (Fig. 1).

**Figure 3:**
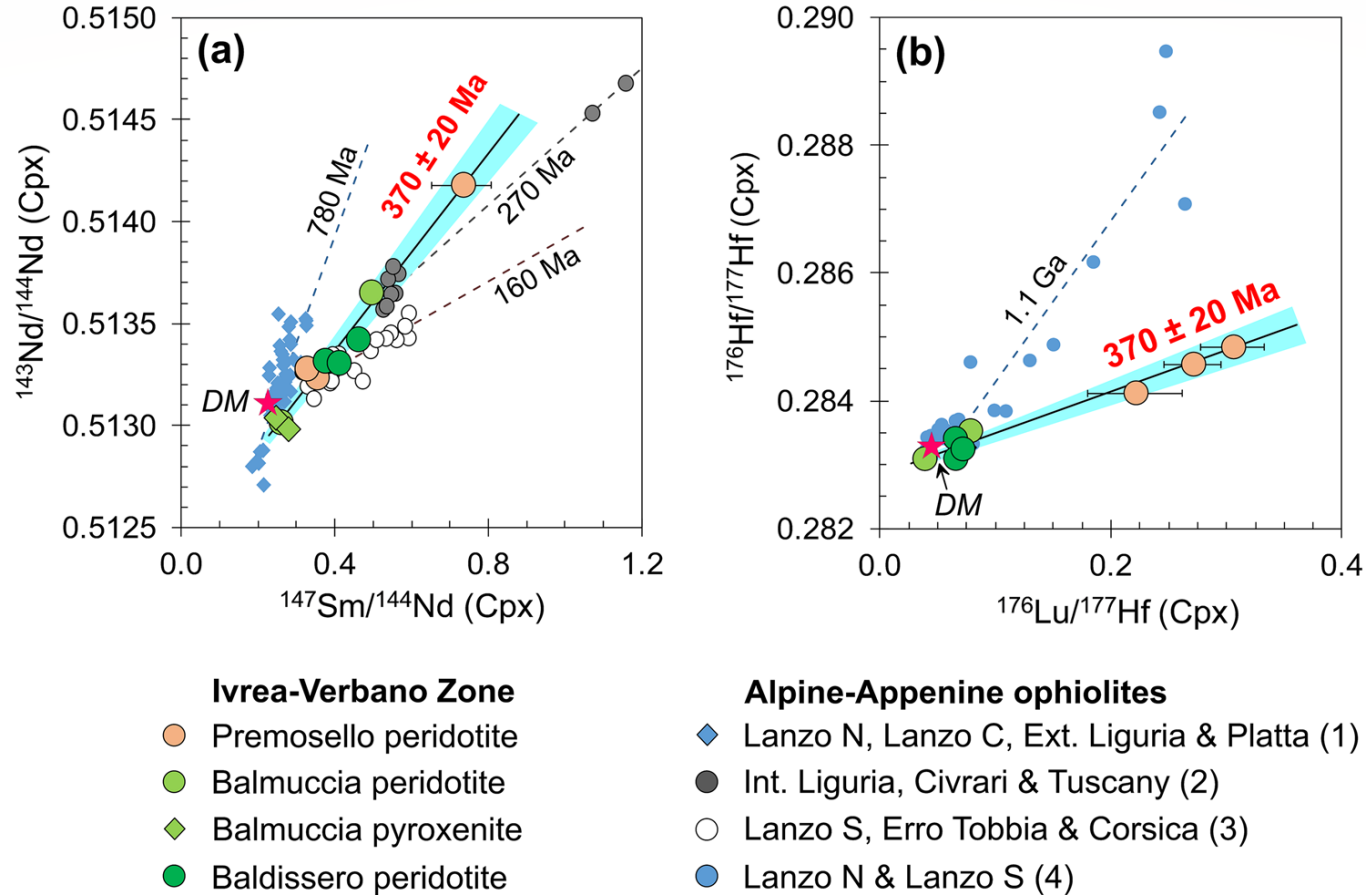
(**a**) Sm/Nd and (**b**) Lu/Hf errorchrons of IVZ lherzolites and pyroxenites (370 ± 20 Ma) compared to errorchrons of Alpine-Apennine ophiolites from (1) Lanzo North, Lanzo Central, External Liguria and Platta^35–38^; (2) Internal Liguria, Civrari and Tuscany^39–41^; (3) Lanzo South, Erro-Tobbio and Corsica^36,42,43^; and (4) Lanzo North and Lanzo South^35,44^. ^143^Nd/^144^Nd and ^176^Hf/^177^Hf error bars are smaller than symbols.

Taken as a whole, the samples from the three mantle massifs preserve well-defined correlations between present-day Nd-Hf isotope ratios and parent-daughter ratios, which are aligned along errorchrons different from those of the Alpine ophiolites (see discussion in Refs ^35–44^). Considering the 2σ errors in ^147^Sm/^144^Nd and ^176^Lu/^177^Hf particularly large for some of the Premosello peridotites, the regression of all our samples return errorchrons of 370 Ma, with an uncertainty of ± 20 Ma. These values are exceptionally consistent for both Sm-Nd and Lu-Hf systematics (Fig. 3). This age is also consistent with Sm-Nd pseudo-isochrons (378 ± 48 Ma, 390 Ma)^28,29^ and peak Paleozoic Re depletion ages (350–500 Ma)^7,25^ previously reported for the Balmuccia and Baldissero lherzolites, and will be later discussed as the timing of the isotopic resetting for both Nd-Hf isotope systematics.

## Discussion

### Isotopic response to Paleozoic mantle melting and chemical re-enrichment

**Figure 4:**
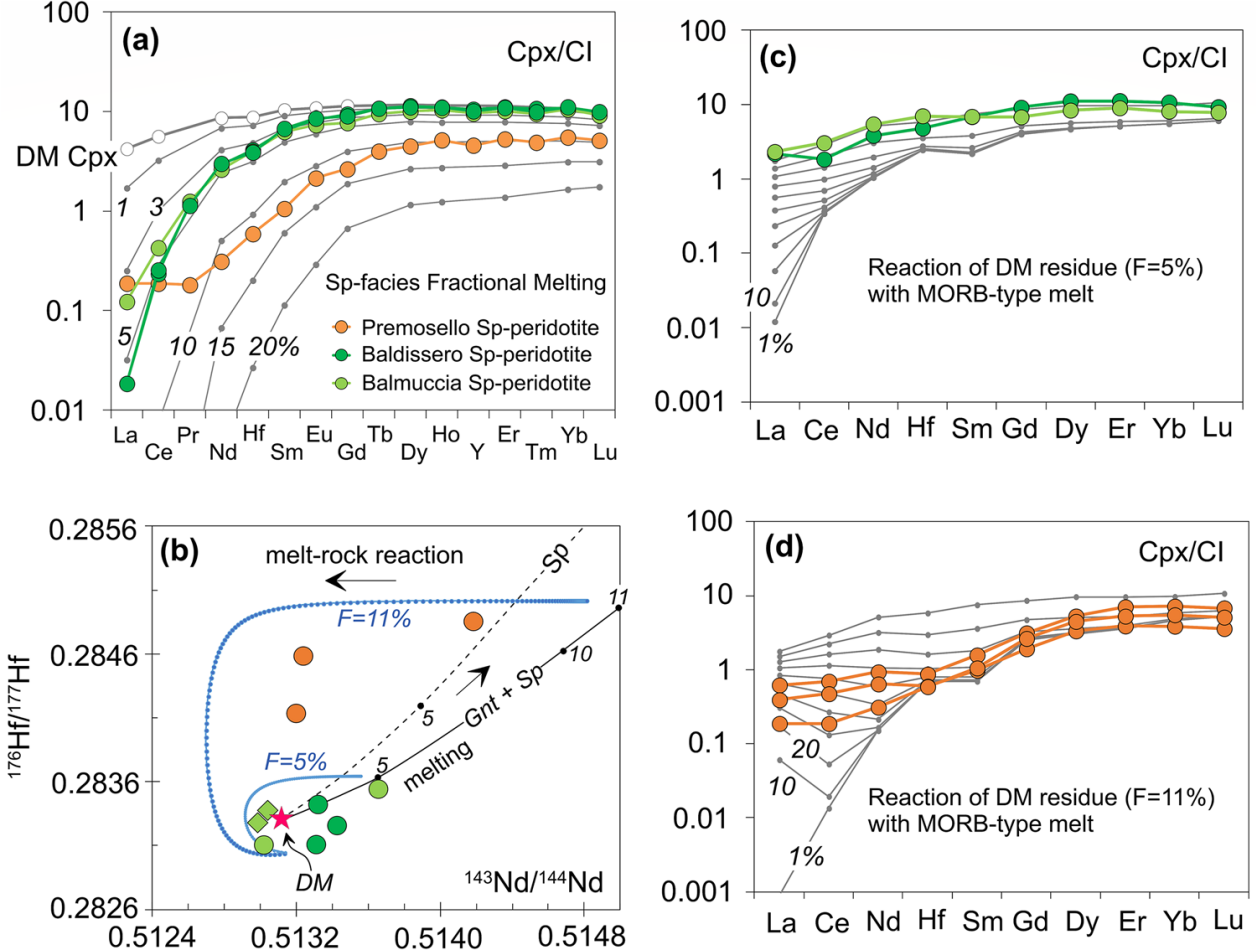
(**a**) Chondrite-normalized REE–Hf patterns of clinopyroxenes (cpx) from the most refractory IVZ lherzolites compared to cpx from DM residues after variable degrees of fractional melting; (**b**) Nd-Hf isotopic evolution of DM residues (bulk rock) after 5, 10 and 11% of partial melting at 370 Ma (values plotted are present day isotope ratios estimated as initial isotope ratios at 370 Ma plus radiogenic ingrowth after 370 Ma). Melting in the garnet + spinel facies and spinel only facies conditions are denoted by unbroken and broken black lines, respectively. The present day isotopic evolution paths of DM residues (F = 5, 11%) after interaction with MORB-type melt at 370 Ma are indicated by blue lines; (**c**-**d**) REE–Hf patterns of cpx from DM residues (F = 5, 11%) after variable degrees of interaction with MORB-type melt (between 1 and 100% re-equilibration, in 10% increments) compared to cpx from refertilized peridotites of this study. Details of the modeling can be found in the methods section and Supplementary Tables S4–S5.

Field, textural and geochemical evidence suggest that the IVZ lherzolites have a complex history of partial melting and melt migration. They show elemental compositions similar to abyssal peridotites (Fig. 2a), and range from residual to melt-reacted. Specifically, cpx from three Balmuccia and Baldissero peridotite samples exhibit strong LREE-depleted patterns consistent with a residual character, and their REE patterns can be reproduced by ~ 5% fractional melting of a DM-like source (Fig. 4a). The other two samples, instead, have LREE too high requiring enrichment by melt addition. In Premosello samples, cpx have low M-HREE and Y reproducible by ~ 10–12% depletion of a DM source. However, all Premosello cpx have LREE too high for only fractional melting to be responsible, and require reaction with melts (Fig. 4a).

Based on the exceptional preservation of Nd and Hf errorchrons giving similar ages, we infer that the resetting of Nd-Hf isotope systematics for all samples occurred at an age of circa 370 Ma (Fig. 3). The two errorchrons do not provide precise age measurements but give temporal constraints to the event of isotopic resetting, which in the case of the IVZ peridotites likely occurred in the middle Paleozoic, specifically in the Upper Devonian. Coherently, Re-Os isotopes of the IVZ peridotites also suggest a major resetting event in the Paleozoic^7,25^.

As suggested by the local preservation of a few Proterozoic Re depletion ages^7^, one hypothesis is that the present-day decoupled, highly radiogenic Nd-Hf isotope compositions of Premosello peridotites were generated by metasomatism at ca. 370 Ma, but the original peridotites were already depleted during an older Proterozoic melting event. Hence, the metasomatism affecting the IVZ peridotites could have been the result of a much younger (c.f., Paleozoic) secondary event in a Proterozoic SCLM (e.g., Refs ^7,45^). If this was the case, however, the samples might not have preserved similar errorchrons in both Nd and Hf systematics, as the highly radiogenic Hf isotopic signature of Premosello cpx would have already existed prior to 370 Ma. There is also the high possibility that ancient mantle metasomatism would have affected ancient isotopically depleted peridotites, mitigating their isotopic and trace element depletion^34^. Moreover, when the Nd-Hf isotopes of all lherzolite cpx samples from the three studied massifs are corrected to 370 Ma, highly radiogenic signatures ascribable to any significant melting event in the Proterozoic was not found. Rather, Premosello peridotites exhibit initial Nd-Hf isotopes (εNd_(i)_ =  + 4.3 to + 6.4, εHf_(i)_ =  + 2.3 to + 7.1) similar to Balmuccia and Baldissero peridotites (εNd_(i)_ =  + 2.9 to + 5.8, εHf_(i)_ =  + 5.5 to + 16.4) suggesting that prior to ca. 370 Ma, the three peridotite bodies had similar isotopic and geochemical compositions. The few unradiogenic ^187^Os/^188^Os isotopes which yielded Proterozoic Re depletion ages in the IVZ lherzolites^7^ may therefore be interpreted as the composition of ancient mantle lithosphere relics delaminated and entrapped in an upwelling asthenosphere. Hence, rather than portions of an ancient depleted (cratonic) SCLM refertilized in more ‘recent’ times, we prefer the possibility that the three peridotite bodies were in the asthenospheric mantle (see also Ref ^46^) and evolved with a similar geochemical composition until ca. 370 Ma.

In line with the preceding statement and supported by isotopic and trace elements modeling (Fig. 4b–d), we hypothesize that at ca. 370 Ma, an intrinsically homogenous asthenospheric mantle section suffered variable degrees of partial melting (i.e., up to 12% in Premosello, ~ 5% in Balmuccia and Baldissero) plus different extents of nearly contemporaneous refertilization which partly obscured the depletion signatures. The original Sm/Nd and Lu/Hf ratios were modified by melting and melt-rock reaction, thereafter the different peridotite bodies evolved by Nd-Hf radiogenic ingrowths along an isotopic evolution dictated by their Sm/Nd and Lu/Hf ratios, in turn defined by both melting and melt-rock reaction processes (Fig. 4b). Hence, the two errorchrons pointing to ca. 370 Ma may represent the age of depletion for both the residual and refertilized peridotites, also coinciding with the timing of the metasomatic event for the refertilized rocks. Notably, the ca. 370 Ma Nd-Hf errorchrons are preserved when the compositions of the pyroxenites are also considered (Fig. 3a,b). Previous studies^24,26^ interpreted this first generation of pyroxenites as segregations of mantle melts with MORB affinity, which also caused refertilization of the host lherzolites. This is in agreement with the MORB-like REE signature of the cpx in our pyroxenite samples, along with their DM-like isotopic compositions. Although the Nd-Hf isotopic compositions of the pyroxenites and the host peridotites might have been rather similar at ca. 370 Ma, migration of such melts modified the parent/daughter ratios causing deviation in isotopic evolutions of the residual vs. the melt-reacted peridotites. This effect is well depicted by the Balmuccia peridotite sample having REE and present-day Nd-Hf isotopes nearly coinciding with the pyroxenites (Figs. 1,2b). This sample interacted extensively with the pyroxenite-forming melt, following a similar isotopic evolution.

### Geodynamic implication and global significance

**Figure 5:**
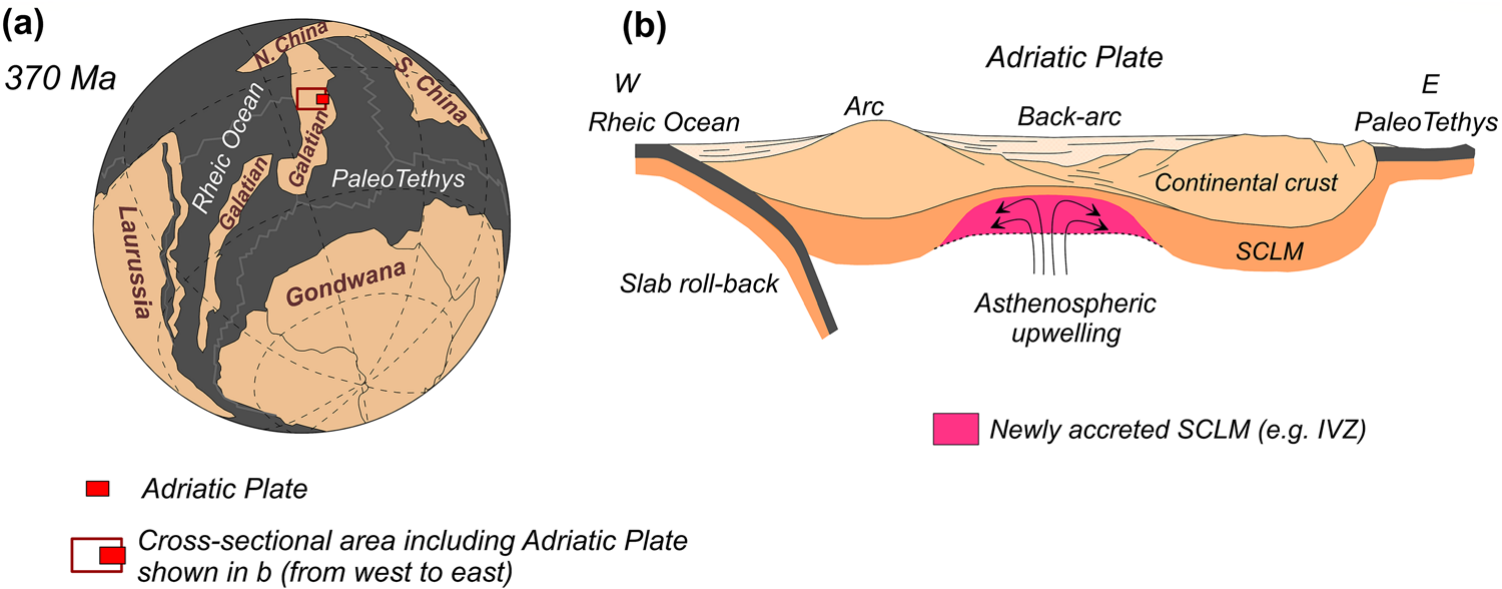
(**a**) Global reconstruction of the Devonian showing the location of Adria within the Galatian terrane (modified after Ref ^48^); (**b**) Schematic model of accretion of the IVZ SCLM by asthenospheric upwelling in the Devonian (adapted from Ref ^49^).

We here report unequivocal Sm-Nd and Lu-Hf isotopic evidence that combined with previously reported Sm-Nd pseudo-isochrons^28,29^ and peak Paleozoic Re depletion ages^7,25^ constrain the accretion of the lherzolitic SCLM beneath the IVZ to the Upper Devonian. At that time, the Adriatic Plate was part of the Galatian terrane (Fig. 5a), a continental ribbon detached from Gondwana and accreted to the margin of Laurussia shortly before the Late Carboniferous Variscan collision^47,48^. At ca. 370 Ma, the northern and western borders of the Galatian terrane were characterized by a long-lasting extension in a back-arc region caused by the subduction of the Rheic Ocean, whereas the southern and eastern ones were passive margins of the PaleoTethys. The lithospheric thinning led to the development of large basins associated with intrabasinal magmatism, starting from 370 Ma and well documented in both Southern Alps and Austroalpine units^49,50^. In this framework, we propose that the IVZ lherzolitic massifs were accreted to the SCLM through asthenospheric upwelling triggered by Variscan intra-continental extension in a back-arc setting related to the subduction of the Rheic Ocean (Fig. 5b). This model of lithospheric accretion in a back-arc environment well explains (i) the low degrees of partial melting inferred for the IVZ peridotites, and the nearly contemporaneous migration of melts refertilizing the peridotites and crystallizing pyroxenites at deep lithospheric levels, (ii) the emplacement of young, fertile lithospheric mantle below older and thinned continental crust, as in the Variscan realm including the IVZ, and (iii) the exhumation of mantle peridotites to crustal levels during compressive regimes affecting thinned back-arc continental lithosphere. Hence, rather than the product of recent processes of rejuvenation of old cratonic roots, we here suggest a model of formation of Phanerozoic SCLM in “recent” continental back-arc settings, where a combination of low-degree melting and nearly contemporaneous melt migration produce fertile mantle lithologies. Young mantle lithosphere thus exists off-craton, even if old Re depletion ages preserve memories of ancient melting events captured during lithospheric accretion.

## Methods

### Mineral major and trace element chemistry

The major element compositions of mineral phases (olivine, orthopyroxene, clinopyroxene and spinel) in selected samples of IVZ lherzolites and pyroxenites were measured by electron probe microanalysis using a JEOL JXA-8230 Superprobe equipped with five WDS spectrometers operating in wavelength dispersive mode, housed at the Joint Laboratory of the Department of Earth Sciences, University of Florence and the CNR-IGG Florence. Operating conditions were 15 kV accelerating voltage, 20 nA beam current, 3 μm spot size, and a counting time of 15 s on the peaks and 7 s on the backgrounds. Natural minerals (olivine for Mg; albite for Si and Na; ilmenite for Fe and Ti; bustamite for Mn; sanidine for K; plagioclase for Al; diopside for Ca; metallic nickel for Ni; chromite for Cr) were used as standards. The results were corrected for matrix effects using the conventional ZAF method provided by the JEOL software package. Results are considered to be accurate and precise within ± 2–5%, as estimated by analysis of natural mineral standards performed during each analytical session. The result of the major element composition of the mineral phases is reported in Supplementary Table S1. The trace element contents of clinopyroxenes were measured on thin sections and mineral separates using an Agilent 8900 QQQ-ICP-MS coupled to a 266 nm Nd:YAG laser ablation system at the CNR-IGG Pavia. The ICP-MS was tuned using NIST SRM 610 synthetic glass to optimize the signal intensity and stability and remove molecular interferences by monitoring ^24^Mg, ^115^In, ^238^U and the ^232^Th/^248^ThO ratio. Data reduction was done with the GLITTER software^51^. The laser was operated at a repetition rate of 10 Hz, fluence of 10 J/cm^2^ and 50–60 μm spot size. NIST SRM 610 was used as an external standard, whereas ^44^Ca was used as internal standard for the clinopyroxene. USGS reference sample BCR2g was repeatedly analyzed together with the unknowns to assess precision and accuracy at ± 5% and ± 10%, respectively. The trace element dataset of the clinopyroxenes is reported in Supplementary Table S2.

### Nd and Hf isotopic measurements

Nd and Hf isotope measurements on clinopyroxene separates from the samples were performed at the National High Magnetic Field Laboratory, Florida State University. For each sample, ~ 100–120 mg of clinopyroxene separates were leached, dissolved, processed through ion exchange columns and measured for Nd-Hf isotopes. The separates were leached in 5 ml 2.5N HCl and < 30% H_2_O_2_ for 60 min at room temperature to remove any alteration products. The leached separates were rinsed several times with quartz sub-boiling distilled water. Subsequent dissolution and column chemistry was performed after procedures described by refs ^52,53^. Nd and Hf isotopes were measured using a ThermoFisher Neptune Multi-Collector ICP-MS system. Measurements of the La Jolla standard yielded ^143^Nd/^144^Nd ratio of 0.511790 ± 0.000012 (2σ, n = 17). The ^143^Nd/^144^Nd ratios were corrected for mass bias using a ^146^Nd/^144^Nd ratio of 0.7219 and are reported relative to the La Jolla standard of 0.511850. Blanks for Nd were less than 10 pg. Measured value of the JMC-475 standard is ^176^Hf/^177^Hf = 0.282150 ± 0.000008 (2σ, n = 20). The ^176^Hf/^177^Hf ratios were corrected for mass bias using a ^179^Hf/^177^Hf ratio of 0.7325 and reported relative to JMC-475 value of ^176^Hf/^177^Hf = 0.282150. Blanks for Hf were less than 40 pg. ^147^Sm/^144^Nd and ^176^Lu/^177^Hf ratios were calculated from the Sm, Nd, Lu and Hf concentrations measured on clinopyroxenes by LA-ICP-MS. The Nd-Hf isotopes dataset of the clinopyroxenes is reported in Supplementary Table S3.

### Partial melting modeling

Modeling of the degree of partial melting underwent by the IVZ lherzolites was performed following the dynamic melting model described in detail by Ref ^54^. The dynamic melting model calculates the trace element and isotopic compositions of mantle residues using the average Depleted Mantle (DM) estimates^13^ as the initial source composition. The choice of the DM end-member as the initial source composition is based on (i) the presumption that the composition of the Upper Mantle (UM) at 370 Ma (according to errorchron ages provided in this study, see Results section) could not have been Primitive, as the UM is expected to have previously experienced about 2–3% partial melting to produce the continental crust (CC) and a complementary DM reservoir (Refs ^13,55^); (ii) the similarity of the initial εNd and εHf of the studied IVZ lherzolites to those of the average DM (εNd_(i)_ =  + 7.9, εHf_(i)_ =  + 16.0)^13^; and (iii) the average lherzolitic modal composition of the DM^55^.

In the modeling, residual porosity was fixed at 1% and percentage of melting per km at 0.15%. Partition coefficients are from Ref ^13^. Garnet melting is assumed to begin at 100 km (degree of melting, F = 0–3.5%) followed by further melting in the spinel stability field. Spinel melting starts at 75 km. Melting reactions are recalculated at 60, 48, 33, and 24 km following the phase relations from Ref ^56^. The present-day isotopic compositions of ancient residues are calculated as initial isotope ratios plus radiogenic ingrowth after 370 Ma. Initial isotope ratios at the time of melting were calculated using a two-stage DM mantle evolution starting from primitive mantle at 3.5 Ga and present-day DM isotope ratios of ^176^Hf/^177^Hf = 0.28330 and ^143^Nd/^144^Nd = 0.51311 from Ref ^13^. Results and starting compositions of the partial melting modeling are reported in Supplementary Table S4.

### Melt-rock reaction modeling

Melt-rock reaction modeling of the IVZ lherzolites was performed following Ref ^34^. We opted for an assimilation-fractional crystallization (AFC) model based on Eqs. 6a and 15a from Ref ^57^. The model reproduces the reaction between a melt and a peridotite. The interaction forms a re-enriched peridotite gradually acquiring a lherzolitic composition by preferential dissolution of olivine and crystallization of clinopyroxene (partition coefficients as in the melting model). This approach follows natural examples of melt-rock reaction in SCLM (e.g., Refs ^12,34,44^). The incoming melt is assumed to crystallize in the matrix and its incompatible element content is redistributed in chemical equilibrium between melt and solid. The trace element composition of the solid is calculated from the liquid following the equation Cs = DC_L_, where D is the bulk partition coefficient for a given element. The trace element composition of the reacted peridotite is calculated at 1% increments, which indicate the mass fraction of peridotite equilibrated with the melt undergoing AFC (scaled from 0 to 100%). Degree of interaction corresponds to ‘F’. We performed several scenarios, using N-MORB and E-MORB starting melts compositions^58^. The use of N- or E-MORB trace element compositions causes subtle differences in the melt-rock reaction trends. Since the Nd-Hf isotopic composition of clinopyroxene from the slightly LREE-depleted peridotite from Balmuccia is similar to the composition of E-MORB, we preferably used an E-MORB-like starting melt composition and mantle residues after 5 and 11 degrees of partial melting (under garnet + spinel conditions) to reproduce the isotopic and trace elements compositions of the refertilized peridotites from Balmuccia and Baldissero (Supplementary Table S5a) and Premosello (Supplementary Table S5b), respectively. The use of mantle residues melted under garnet + spinel facies conditions for the melt-rock reaction is mainly due to the plotting of the studied Balmuccia and Baldissero peridotite cpx samples around the garnet + spinel melting evolution line (as shown in Fig. 4b) and the MREE/HREE ratios of these cpx being steeper than those produced by spinel only fractional melting suggesting deeper melt depletion for the IVZ lherzolites (as also earlier documented by Ref ^25^). Present-day isotopic compositions are calculated adding the initial isotope ratios, calculated from Eq. (15a) from Ref ^57^, to the radiogenic Nd and Hf ingrowth after 370 Ma.

### Supplementary Information

Supplementary Information.

Supplementary Tables.

## Data Availability

All analytical data and additional figures reported in this paper can be found in the accompanying Supplementary Information files (Tables S1–S5 and Figs. S1–S4).
