# Supplementary material for: Accretion of “young” Phanerozoic subcontinental lithospheric mantle triggered by back-arc extension—the case of the Ivrea-Verbano Zone
Source: Sci Rep. 2024 May 23;14:11805. doi: 10.1038/s41598-024-61763-3 (PMC11116441; doi:10.1038/s41598-024-61763-3)
Supplement: Supplementary file 1 — Supplementary Information. [file 41598_2024_61763_MOESM1_ESM.pdf]

SUPPLEMENTARY INFORMATION FOR

Accretion of "young" Phanerozoic subcontinental lithospheric mantle triggered by back-arc extension – the case of the Ivrea-Verbano Zone

Abimbola C. Ogunyele[1,2,3], Alessio Sanfilippo[1,2,*], Vincent J. M. Salters[4], Mattia Bonazzi[2] and Alberto Zanetti[2]

[1] Department of Earth and Environmental Sciences, University of Pavia, Via Ferrata 1, 27100 Pavia, Italy

[2] CNR – Istituto Geoscienze e Georisorse, Via Ferrata 1, 27100 Pavia, Italy

[3] Department of Earth Sciences, Adekunle Ajasin University, PMB 001 Akungba-Akoko, Nigeria

[4] National High Magnetic Field Laboratory, Department of Earth, Ocean and Atmospheric Sciences, Florida State University, Tallahassee, FL 32310, USA

*correspondence to alessio.sanfilippo@unipv.it

Table of contents

Figure S1. Geological settings of the Ivrea-Verbano Zone (IVZ) and the locations of studied orogenic peridotite massifs

Figure S2. Field and petrographic description of the Balmuccia peridotite massif

Figure S3. Field and petrographic description of the Baldissero peridotite massif

Figure S4. Field and petrographic description of the Premosello peridotite massif

Table S1. Major element composition of mineral phases from IVZ lherzolites and pyroxenites

Table S2. Trace element composition of clinopyroxenes from IVZ lherzolites and pyroxenites

Table S3. Nd-Hf isotopic composition of clinopyroxenes from IVZ lherzolites and pyroxenites

Table S4. Results of partial melting modeling

Table S5. Results of melt-rock reaction modeling

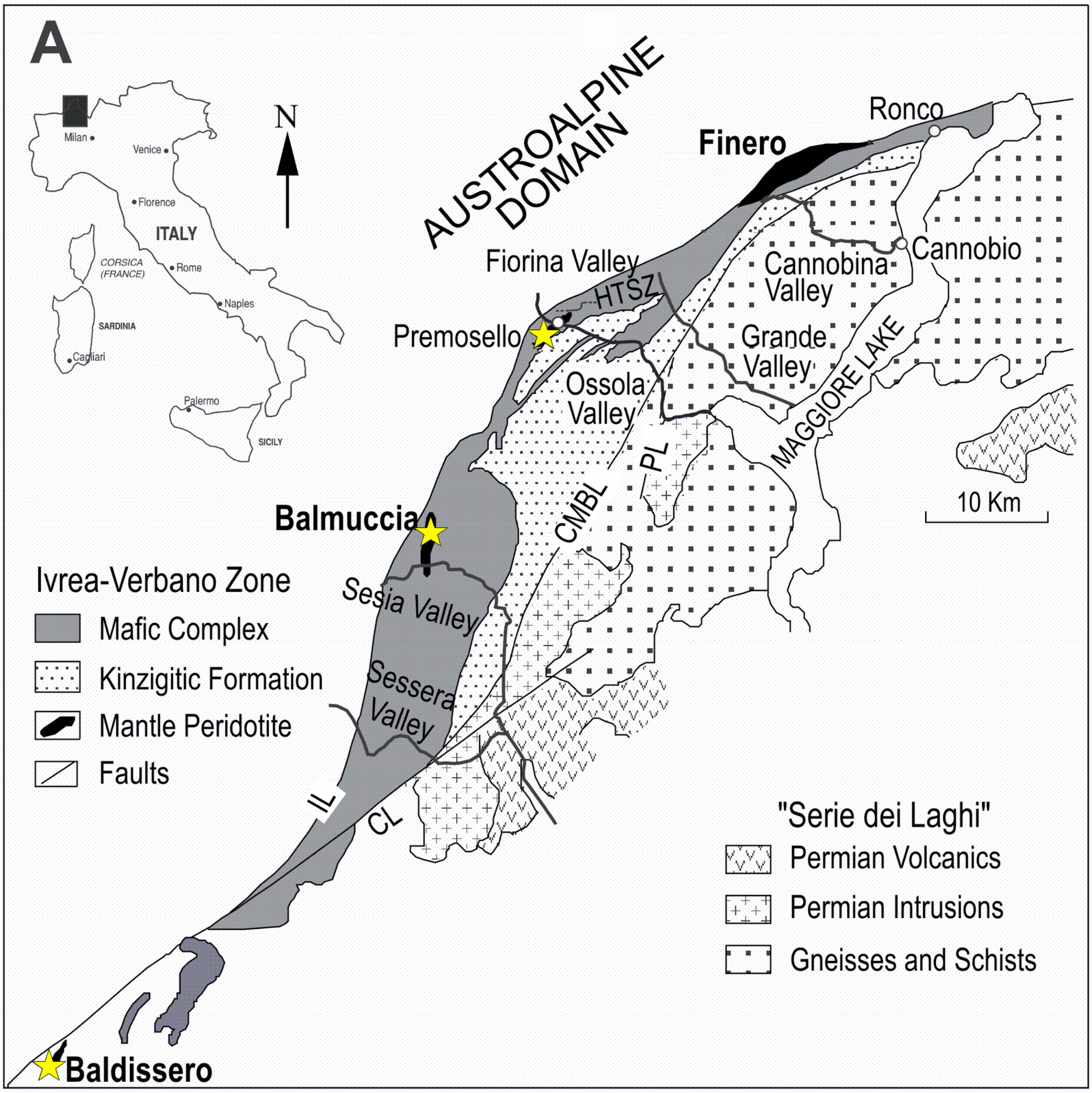

Figure S1. Geological settings of the Ivrea-Verbano Zone and the locations of studied orogenic peridotite massifs at Balmuccia (45.816853 N, 8.136250 E), Baldissero (45.42098 N, 7.75106 E) and Premosello (46.005298 N, 8.320076 E). IL – Insubric Line, CMBL – Cossato-Mergozzo-Brissago Line, CL – Cremosina Line, PL – Pogallo Line, HTSZ – High Temperature Shear Zone (after Zanetti et al., 1999; Mazzucchelli et al., 2010).

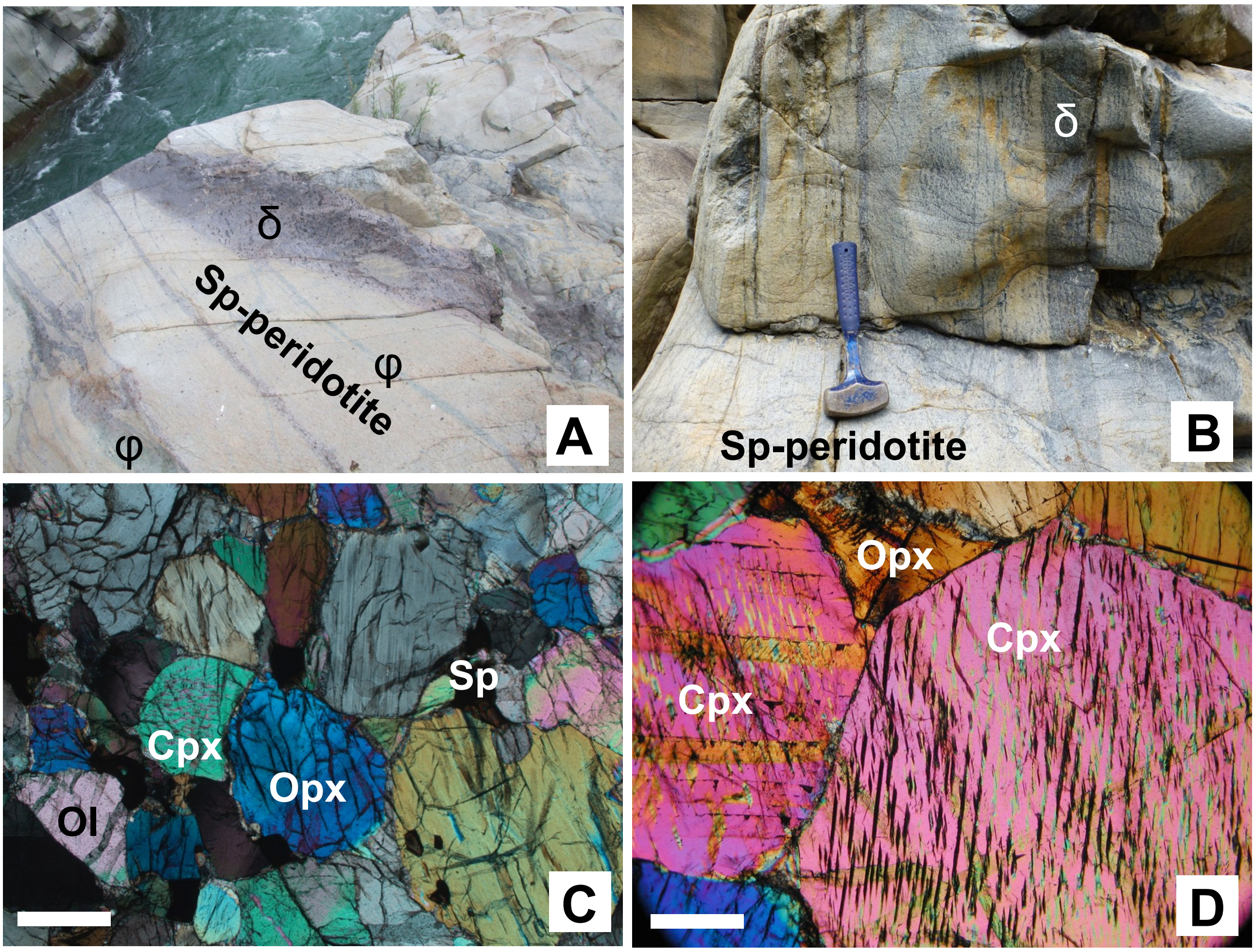

Figure S2. (A, B) Spinel lherzolite of the Balmuccia mantle massif cut by Cr-diopside clinopyroxenite (φ) and Al-augite pyroxenite (δ); (C) thick sections (60 μm, x-nicols) of Balmuccia lherzolite showing protogranular texture and (D) Cr-diopside websterite displaying granoblastic texture with 120° triple junctions. The white bars to the left of panels (C, D) represent 1 mm scale.

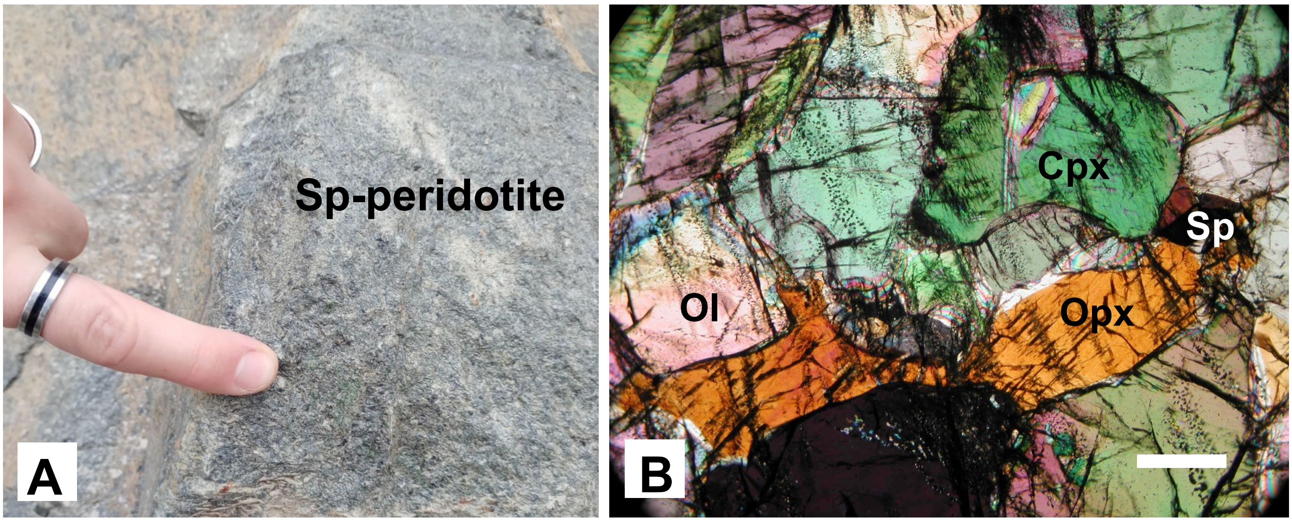

Figure S3. (A) Spinel lherzolite of the Baldissero mantle massif; (B) thick section (60 µm, x-nicols) of Baldissero lherzolite showing protogranular texture. The white bar to the right of panel (B) represents 1 mm scale.

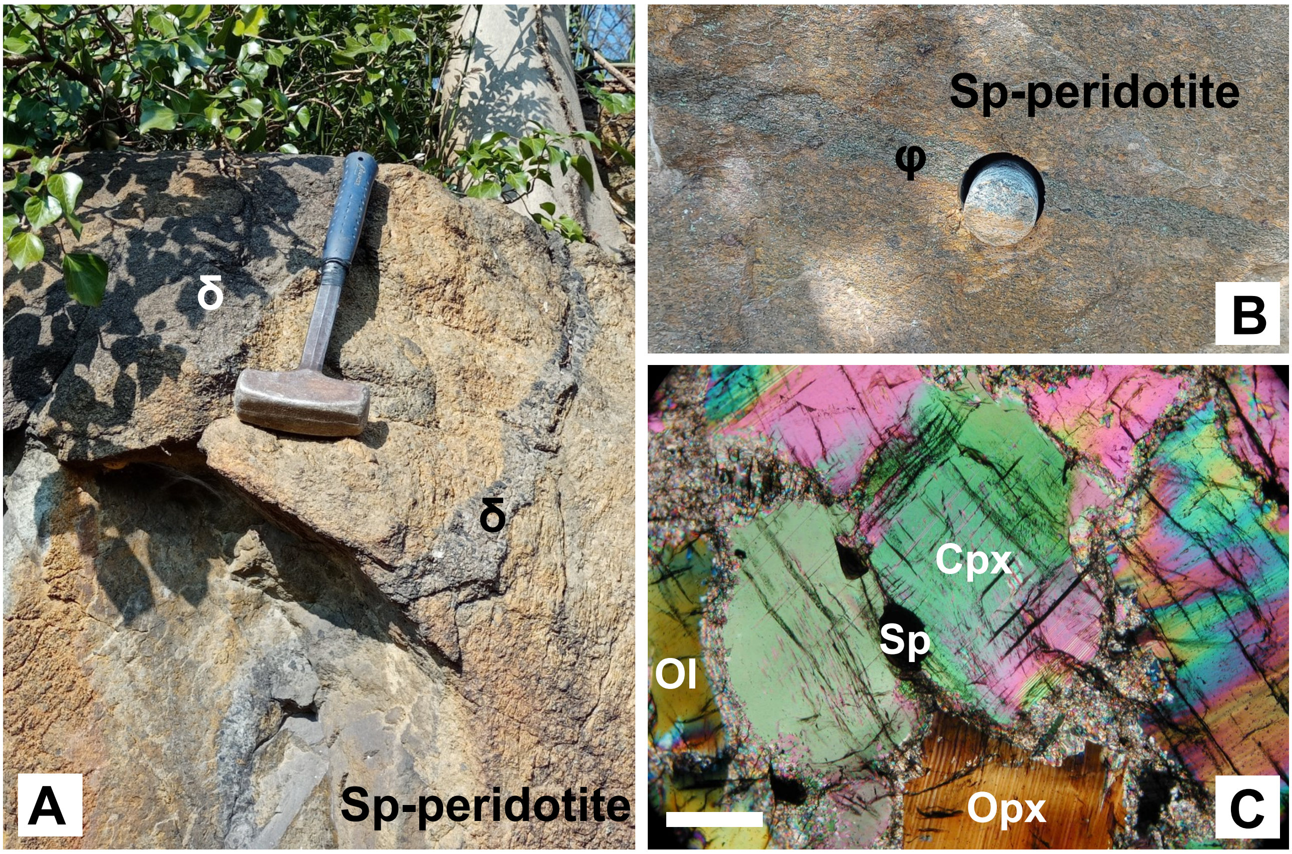

Figure S4. (A, B) Spinel lherzolite of the Premosello mantle massif cut by Cr-diopside clinopyroxenite (φ) and Al-rich diopside pyroxenite (δ); (C) thick section (60 µm, x-nicols) of Premosello lherzolite showing porphyroclastic texture. The white bar to the left of panel (C) represents 1 mm scale.

References

Mazzucchelli, M. *et al*. Age and geochemistry of mantle peridotites and diorite dykes from the Baldissero body: Insights into the Paleozoic-Mesozoic evolution of the Southern Alps. *Lithos* **119**, 485–500, https://doi.org/10.1016/j.lithos.2010.08.002 (2010).

Zanetti, A., Mazzucchelli, M., Rivalenti, G. & Vannucci, R. The Finero phlogopite–peridotite massif: an example of subduction-related metasomatism. *Contrib. Mineral. Petrol.* **134**, 107–122, https://doi.org/10.1007/s004100050472 (1999).
